# Morphological and molecular analyses of season-specific responses of freshwater ciliate communities to top-down and bottom-up experimental manipulations

**DOI:** 10.1128/msystems.00304-25

**Published:** 2025-08-15

**Authors:** Usman Asghar, Indranil Mukherjee, Bettina Sonntag, Caio César Pires de Paula, Vojtěch Kasalický, Paul-Adrian Bulzu, Anusha Priya Singh, Tanja Shabarova, Kasia Piwosz, Karel Šimek

**Affiliations:** 1 Biology Centre of the Czech Academy of Sciences, Institute of Hydrobiology 90801 , Ceske Budejovice, Czechia Republic; 2 Faculty of Science, University of South Bohemia, České Budějovice, Czech Republic; 3 Research Department for Limnology, Mondsee, Universität Innsbruck 27255, Salzburg, Austria; 4 National Marine Fisheries Research Institute 123876 , Gdynia, Poland; CNRS Delegation Alpes, Lyon, France

**Keywords:** ciliates, aquatic food web, microbial loop, experimental manipulations, freshwater reservoir, quantitative protargol staining, long-read amplicon sequencing

## Abstract

In aquatic microbial food webs, ciliates represent an important trophic link in the energy transfer from prokaryotes, algae, and heterotrophic nanoflagellates (HNFs) to higher trophic levels. However, the trophic role of abundant small ciliates (<20 µm) is not clearly understood. To unveil their trophic linkages, we conducted two experiments manipulating both top-down and bottom-up controlling factors, thus modulating the trophic cascading and bacterial prey availability for protists during contrasting spring and summer seasons with samples collected from a freshwater meso-eutrophic reservoir. Water samples were size fractionated to modify food web complexity, i.e., 10 µm, 20 µm, and unfiltered control and amended with bacterial prey additions. The samples were analyzed by morphological and sequencing techniques. The bacterial amendments triggered strong ciliate growth following the peaks of HNFs in the 10 and 20 µm treatments, reflecting a trophic cascading from HNFs to raptorial prostome ciliates (*Balanion planctonicum* and *Urotricha* spp.) in spring. In summer, HNFs and ciliates peaked simultaneously, suggesting the important trophic cascade also from bacteria to bacterivorous scuticociliates (*Cyclidium glaucoma* and *Cinetochilum margaritaceum*) and HNFs. In spring, unfiltered treatments showed stronger ciliate top-down control by zooplankton than in summer. The sequence analysis revealed season-specific manipulation-induced shifts in ciliate communities and their large cryptic diversity. However, morphological and molecular analyses also revealed considerable discrepancies in the abundance of major ciliate taxa. The ciliate communities responded to our experimental manipulations in season-specific fashions, thereby highlighting the different roles of ciliates as an intermediate trophic link between prokaryotes and higher trophic levels.

**IMPORTANCE:**

Ciliates represent an important trophic link in aquatic microbial food webs. In this study, we used the food web manipulation techniques to reveal their complex trophic interactions during seasonally different plankton scenarios occurring in spring and summer. Manipulating top-down controlling factors (grazing pressure of micro- and metazooplankton grazers) and bottom-up factors (an availability of bacterial prey) shaped distinctly the complexity and dynamics of natural plankton communities and thus yielded significant changes in ciliate community dynamics. The experimentally simplified plankton and ciliate communities responded to our manipulations in season-specific fashions, reflected in different roles of ciliates as an intermediate trophic link between prokaryotes and higher trophic levels. This study also demonstrates that the combination of molecular and morphological analyses is essential to gain more realistic insights into the structure of ciliate community and for providing robust, ecologically meaningful results.

## INTRODUCTION

Ciliates are an extremely diverse and ubiquitous group of unicellular eukaryotes in aquatic habitats (1, 2), regarding cell size, shape, and trophic roles. Considering these characteristics, the concept of microbial loop (3), attributed to ciliates a key position in the aquatic microbial food webs, as they contribute significantly to the trophic complexity by linking different trophic levels of the food webs (4–7). For instance, ciliates possess various feeding modes allowing them to graze on detritus, prokaryotes, unicellular eukaryotes, other ciliates, and diatoms and are preyed upon by meta-zooplankton, fish larvae, and planktivorous fish (8, 9).

The microbial community dynamics include relative abundance, structure, and composition of prokaryotic and eukaryotic microbes. It changes with the availability of food resources, community structure of zoo- and phytoplankton, and shifts in controlling physical factors (e.g., solar irradiation, temperature, etc.). Based on these patterns, the recurrent seasonal cycles in temperate freshwater ecosystems were divided into four distinct phases: spring phytoplankton bloom, clearwater phase, summer/autumn, and winter (10, 11). This plankton seasonality is also reflected (5, 12–15) in recurring successional patterns in both community composition and abundance of planktonic ciliates in temperate lakes. According to the description of the revised Plankton Ecology Group (PEG) model (11), and of other recent studies (16, 17), ciliated protists are the first and the most voracious grazers of phytoplankton during spring bloom events. The ciliate community during spring is dominated by medium-sized (<30 µm) algivorous oligotrichs and small (>20 µm) prostomatids (*Balanion planctonicum* and *Urotricha* spp.), which are usually replaced first by omnivorous/mixotrophic ciliates and then by small bacterivorous scuticociliates (18).

Additionally, the ciliate community composition and relative abundance of particular species are also influenced by the trophic status of respective lakes. In mesotrophic and meso-eutrophic lakes, the peak in the abundance of ciliates generally occurs during the spring phytoplankton bloom and again in summer (10, 14, 18–21). In contrast, in eutrophic lakes, the seasonal maxima are recorded in late summer since, in spring, ciliates experience a very strong top-down control by zooplankton predators (5, 10, 18). Generally, the ciliate assemblage in spring consists mainly of larger herbivorous ciliate species of the class Spirotrichea (*Pelagostrombidium* spp., *Tintinnidium fluviatile*, and *Codonella cratera*) (20), whose dominance contributes significantly to the total ciliate biomass. Especially in mesotrophic and meso-eutrophic systems, small algivorous Prostomatea (e.g., *B. planctonicum*) show a steep increase in abundance after a bloom of flagellated algae and contribute about 60%–80% to the total ciliate count during their spring maximum (16, 22, 23). The spring maximum of algivorous ciliates is usually followed by the succession of several mixotrophic ciliates (*Pelagohalteria viridis*, *Histiobalantium bodamicum*, *Limnostrombidium viride, Askenasia* spp., and *Mesodinium* spp.) and heterotrophic algivorous *Urotricha* spp. (10, 18), while omnivorous and bacterivorous ciliate species (*Halteria grandinella* and *Rimostrombidium* spp., *Cyclidium* spp., *Uronema* spp.) prevail during a summer/autumn period (20, 24).

Both food availability (bottom-up) and predation by larger zooplankton (top-down) have been suggested as the main factors controlling the abundance and community composition of ciliates in freshwater lakes (7, 13, 25). However, most of the previous studies have analyzed the effects of top-down and bottom-up processes in highly complex natural food webs, where numerous other confounding factors, such as solar irradiation, temperature, pH, oxygen, predation, food quality, etc., influence the structuring of natural plankton communities (11). This considerably limits our ability to distinguish the true causal relationships and to refine our knowledge on the trophic role of the key members of planktonic ciliates.

**Fig 1:**
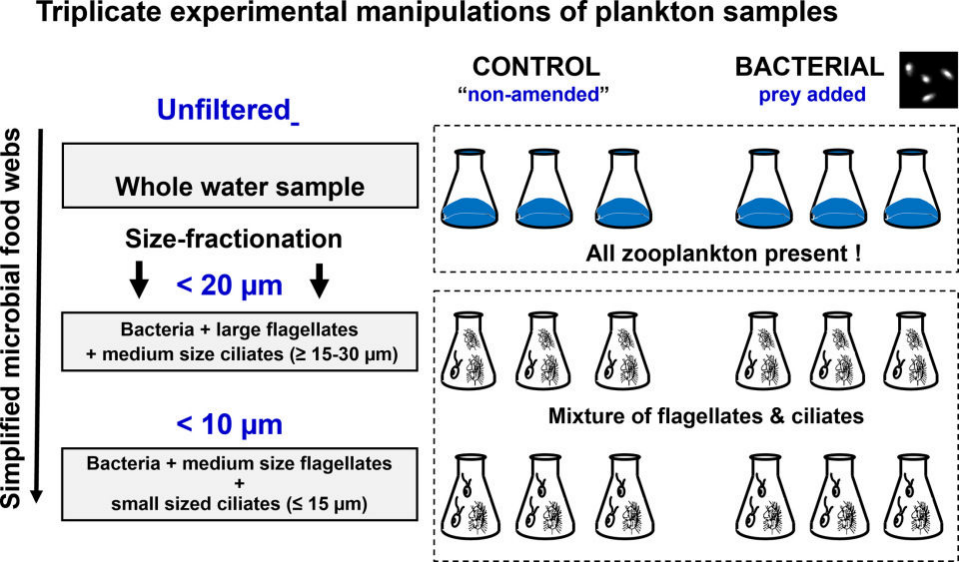
The experimental design showing the treatments: unfiltered—natural microbial and zooplankton communities, 20 µm filtered—supporting growth of micro-sized ciliates and large flagellates, and the 10 µm filtered—supporting growth of nano-sized ciliates and small flagellates.

In this study, we examined the responses of natural ciliate communities, originating from the contrasting spring and summer plankton phases in Římov reservoir, Czech Republic, to experimental manipulations substantially modulating both top-down and bottom-up controlling factors. The plankton samples from the seasonally distinct phases were subjected to the same experimental manipulations, in which we significantly increased bacterial prey availability for protistan communities and, in parallel, we simplified a food web structure and trophic interactions by size fractionation (10 µm, 20 µm, and unfiltered) of the plankton samples (Fig. 1). This resulted in different levels of food web complexity brought about by simplifying the complex natural zooplankton communities as top predators in the unfiltered fraction in contrast to considerably simplified interactions of largely protist-dominated communities in the 20 and 10 µm fractions.

## RESULTS

The environmental parameters from the samples taken from the Římov reservoir in spring and summer are shown in Table 1. In general, based on both quantitative protargol staining method (QPS) and sequencing data, the comparison of ciliate communities showed that the ciliate assemblage was more diverse in summer than in spring. Shannon diversity index also showed significantly higher diversity in the summer (3.36 ± 0.62) as compared with spring samples (2.88 ± 0.54) (File S1; Fig. S1). We were able to detect 18 species of ciliates in spring and 20 in summer using QPS (Table 2). Taxonomically, we found a greater diversity in the class Spirotrichea in spring (44% of the total assemblage), while in summer, the Oligohymenophorea were more diverse (30%), followed by Spirotrichea (25%).

**TABLE 1:** Physical and chemical parameters at the sampling site during spring and summer

Parameter	Spring	Summer
Sampling depth (m)	0.5	4.0
Temperature (°C)	9.6	19.0
pH	6.1	6.8
DO (mg L^−1^)	13.0	7.5
O_2_ Saturation (%)	114.7	85.4
TOC (mg L^−1^)	4.9	8.3
Chl-*a* (µg L^−1^)	15.1	14.2
Total nitrogen (mg L^−1^)	1.6	1.5
Dissolved phosphorus (µg L^−1^)	30.6	24.0

**TABLE 2:** Ciliate taxa detected by QPS during food web manipulation experiments^*a*^

Class	Sub-class	Order	Species	UNF	10 µm	20 µm
Spirotrichea	Choreotrichia	Choreotrichida	*Rimostrombidium humile*	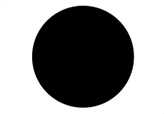	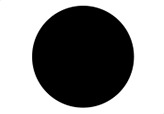	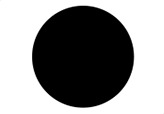
			*Rimostrombidium lacustris*	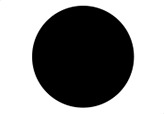		
			*Rimostrombidium brachykinetum*	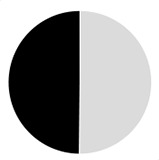		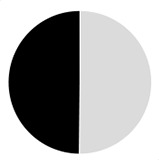
		Tintinnida	*Tintinnidium pusillum*	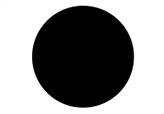		
			*Codonella cratera*	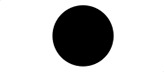		
	Oligotrichia	Strombidiida	*Limnostrombidium viridis*	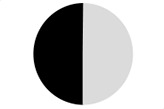		
	Stichotrichia (Hypotrichia)	Sporadotrichida	*Halteria grandinella*	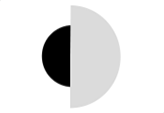		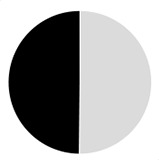
			*Halteria sp*.	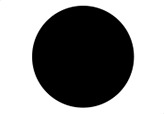	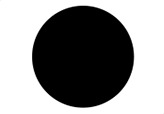	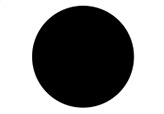
		Stichotrichida	*Stichotricha aculeata*	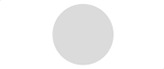		
		Urostylida	*Uroleptus willii*	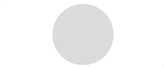		
Prostomatea	**–**	Prorodontida	*Balanion planctonicum*	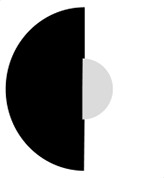	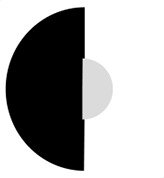	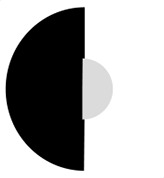
			*Urotricha globosa*	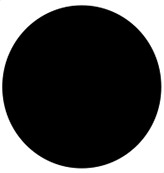		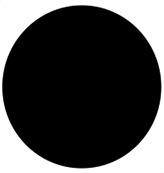
			*Urotricha furcata*	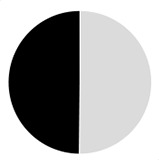	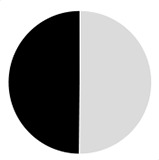	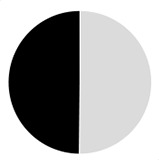
			*Coleps viridis*	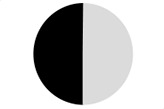	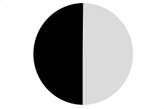	
			*Coleps hirtus*	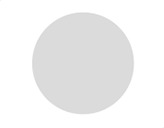	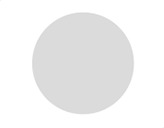	
Oligohymenophorea	Peritrichia	Sessilida	*Vorticella natans*	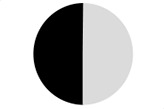	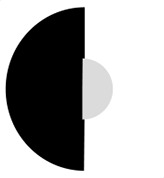	
			*Vorticella aquadulcis complex*	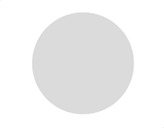		
			*Epistylis procumbens*	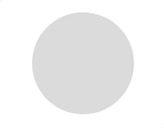		
	Sucticociliata	Philasterida	*Cinetochilum margariticeum*	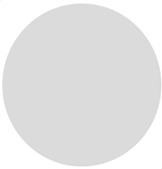		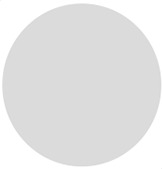
			*Pseudocohnilembus pusillus*	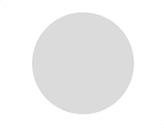		
		Pleuronematida	*Cyclidium glaucoma*	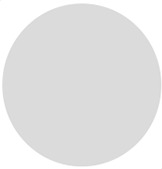	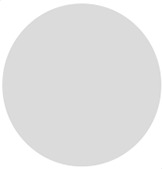	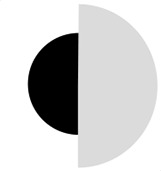
			*Histiobalantium bodamicum*	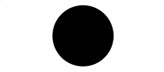		
	Peniculia	Peniculida	*Stokesia vernalis*	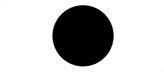		
Litostomatea	Haptoria	Cyclotrichiida	*Mesodinium pulex*	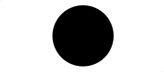	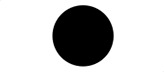	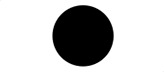
			*Mesodinium perrieri*	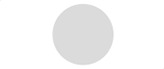		
			*Mesodinium acarus*	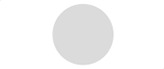	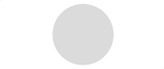	
			*Askenasia chlorelligera*	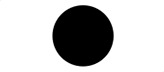	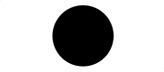	
		Haptorida	*Pelagolacrymaria rostrata*	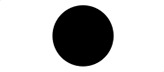		
			*Didinium nasutum*	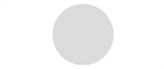		
Colpodea	**–**	Cyrtolophosidida	*Cyrtolophosis mucicola*	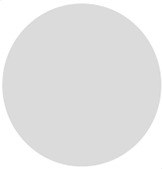	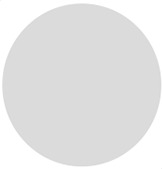	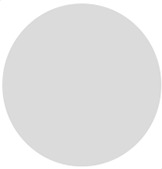
Karyorelictea	**–**	Loxodida	*Loxodes magnus*	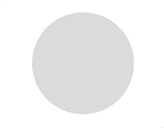		

^*a*^ In different seasons and size fractions. Bubble color denotes seasonal variation ( 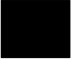 spring 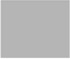 summer), while bubbles size indicates the relative abundance, (high
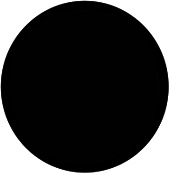
 medium
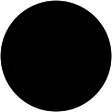
 low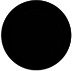 ), (-) is used for ciliate species that lack a subclass-level taxonomic classification.

### Response of the spring ciliate community to experimental manipulations: results from microscopic analyses

**Fig 2:**
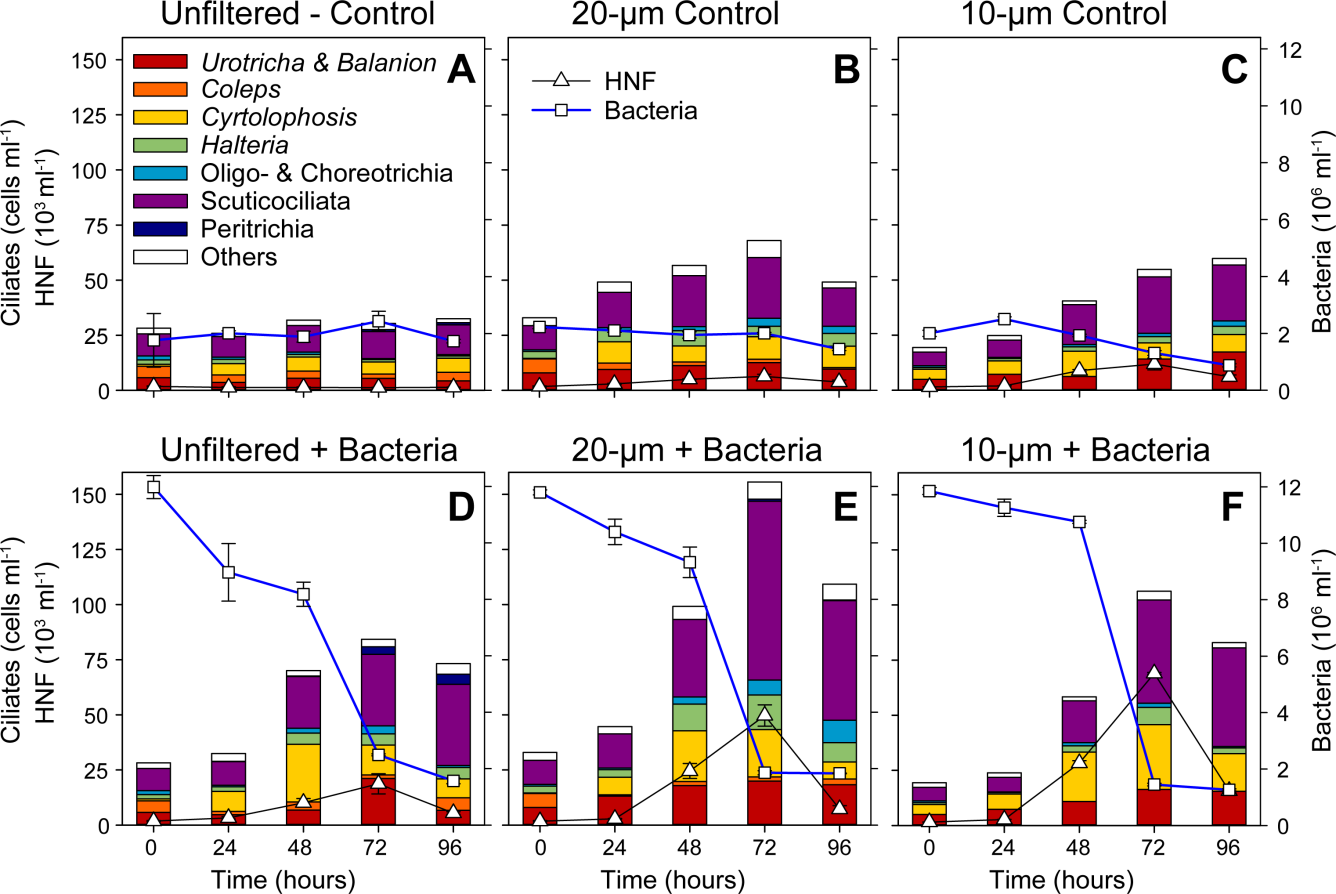
The spring experiment: temporal changes in the abundance of bacteria, HNF and ciliates, and the compositional shifts in the ciliate community (by QPS) between 0 and 96 h. Error bars represent the mean values of abundance of different ciliate taxa. Upper panels show the controls (A–C), lower ones the bacterial amended treatments (D–F), from left to right the unfiltered (**A, D**), the 20 µm (**B, E**) and the 10 µm treatments (**C, F**) are shown. Note: The groups showing higher than the genus level classification in the figure legend are represented by the following genera: Choreotrichia (*Rimostrombidium*, *Tintinnidium*, *Codonella*), Oligotrichia (*Limnostrombidium*), Peritrichia (*Vorticella*), Scuticociliata (*Cyclidium*). For more details, see Table 2.

The size fractionation approach reduced the initial ciliate abundance from the unfiltered (15.1 cells mL^−1^) to the 20 µm (12.5 cells mL^−1^) and 10 µm (9.3 cells mL^−1^) treatments and affected the ciliate community composition (Fig. 2A through F). At T0 h, in the unfiltered treatment, peritrichous *V. natans*, choreotrichous *C. cratera,* and prostomatid *Urotricha* spp. were most abundant, while, in the 20 µm treatment, *Urotricha* spp. and *V. natans*, and in the 10 µm treatment *Urotricha* spp., stichotrichous *H. grandinella* and prostomatid *B. planctonicum* prevailed (Fig. 2; Table 2).

Time-course data from the unfiltered treatment indicated that ciliate communities nearly collapsed both in the control and bacteria-amended treatments at the end of the experiment (Fig. 2A and D). However, we observed the marked growth response (19 × 10^3^ cells mL^−1^) of heterotrophic nanoflagellates (HNFs) at T72 h in the bacteria-amended unfiltered treatment (Fig. 2D). In the 20 µm and 10 µm control treatments, a trend in increasing ciliate abundance was observed with 100.9 cells mL^−1^ and 83.2 cells mL^−1^, respectively (Fig. 2B and C). The prostomatid flagellate grazers *Urotricha* spp. and *B. planctonicum* dominated the treatments.

Notably, in bacteria-amended treatments, HNFs reached their maxima (34.1 × 10^3^ cells mL^−1^) at T48 h in the 20 µm treatment (Fig. 2E), and at T72 h in the 10 µm treatment (85.8 × 10^3^ cells mL^−1^, Fig. 2F). However, the bacteria were rapidly decimated from 13.2 × 10^6^ cells mL^−1^ to only 1.1 × 10^6^ cells mL^−1^ in 20 µm treatment (Fig. 2E). This likely reflected not only the HNF bacterivory but also the possible contribution of peritrichous *bacterivore - V. natans,* which peaked at T72 h (Fig. 2E).

In both the bacteria-amended 10 µm and 20 µm treatments, HNF peaks were followed by conspicuous peaks in ciliate abundance with 186.5 cells mL^−1^ and 182.5 cells mL^−1^, respectively, at T96 h. This sharp increase was mainly due to the rapid growth of prostomatid raptorial flagellate hunters, *B. planctonicum* (10 µm: 81.9 cells mL^−1^, 20 µm: 45.3 cells mL^−1^) and *Urotricha* spp. (10 µm: 80.9 cell mL^−1^, 20 µm: 70.3 cells mL^−1^) (Fig. 2E and F). However, in the 20 µm treatment, the ciliate assemblage was more diverse, along with remarkable growth of bacterivorous peritrichous and choreotrichous ciliates (Fig. 2E). Similarly, results of the ANOVA also revealed that time (F = 77.623, *P* < 0.001), size fractionation (F = 30.514, *P* < 0.001), and prey amendment (F = 9.083, *P* = 0.007) had significant effects on ciliate abundance during spring.

### Response of summer ciliate community to experimental manipulation: results from microscopic analyses

**Fig 3:**
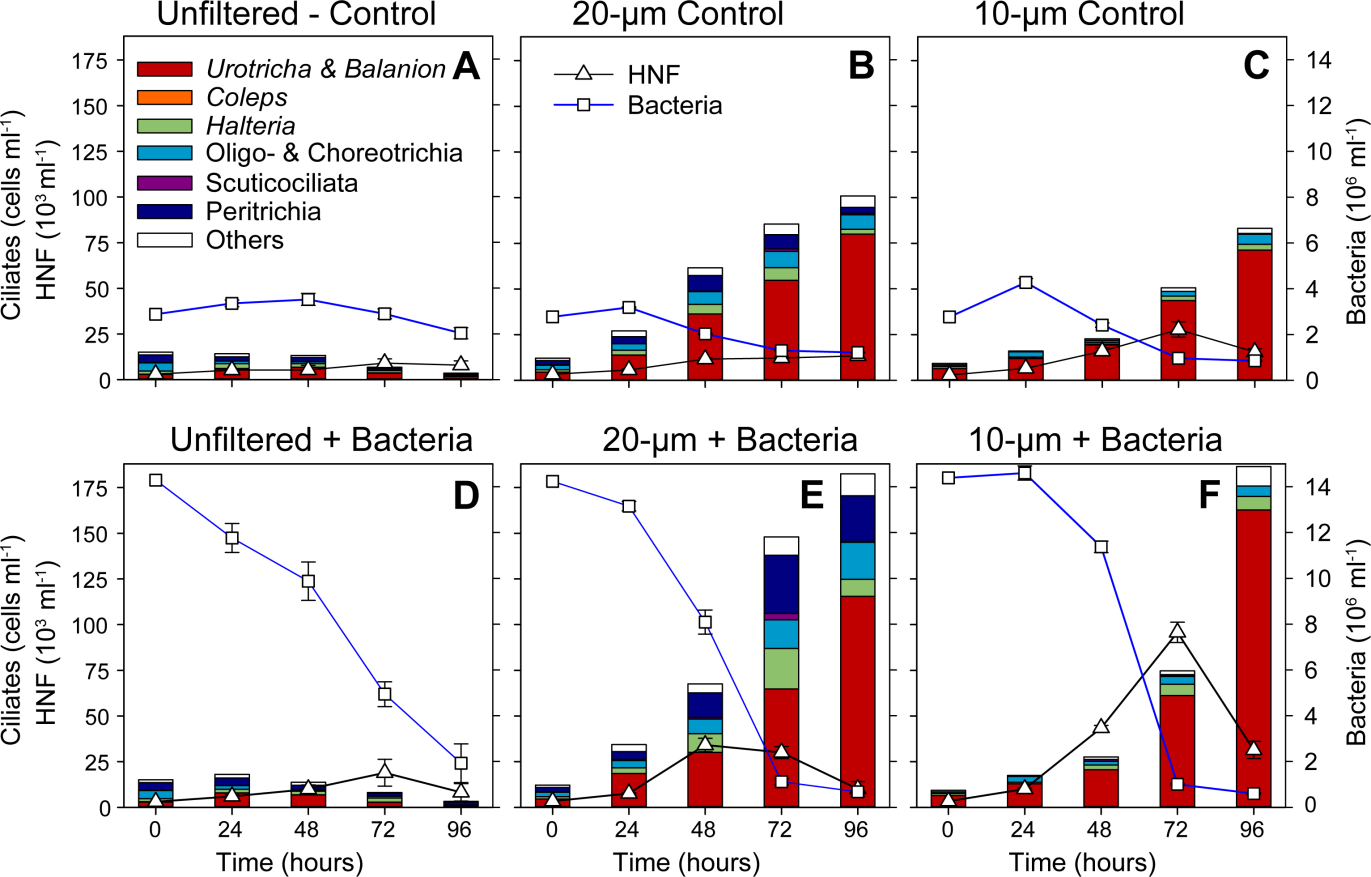
The summer experiment: Temporal changes in the abundance of bacteria, HNF, and ciliates, and the compositional shifts in the ciliate community (by QPS) between 0 and 96 h. Error bars represent the mean values of abundance of different ciliate taxa. Upper panels show the controls (A–C), lower ones the bacterial amended treatments (D–F), from left to right the unfiltered (**A, D**), the 20 µm (**B, E**), and the 10 µm treatments (**C, F**) are shown. Note: The groups showing higher than the genus level classification in the figure legend are represented by the following genera: Choreotrichia (*Rimostrombidium*), Oligotrichia (*Limnostrombidium*), Peritrichia (*Vorticella*, *Epistylis*) Scuticociliates (*Cinetochilum*, *Cyclidium*) for more details, see Table 2.

During summer, the ciliate community was largely dominated by small ciliates. Therefore, the size fractionation yielded similar initial ciliate abundances in the unfiltered (28.3 cells mL^−1^) and the 20 µm (32.7 cells mL^−1^) treatments (Fig. 3A and B), where both the treatments were dominated by prostomatid *Urotricha* spp., *Coleps* spp., and the sucticociliates *C. glaucoma* and *C. margaritaceum* at T0 h (Fig. 3A and B). However, in 10 µm ciliate abundance decreased considerably at T0 h (19.4 cells mL^−1^, Fig. 3C), being accompanied also by shifts in the composition of ciliates towards tiny species, such as dominant *C. glaucoma,* followed by *C. mucicola* (colpodea) and *Urotricha* spp. (Fig. 3C).

In the unfiltered treatments, the ciliate community remained relatively stable with a slight increase in ciliate numbers (Fig. 3A). In bacterial amended treatment, the ciliate reached 81.3 cells mL^−1^ at T72 h, mainly due to increase of sucticociliates *C. margaritaceum* (19.5 cells mL^−1^) and prostomatid *Urotricha* spp. (20.1 cells mL^−1^) (Fig. 3D). Notably, in the summer prey-amended treatments, maxima of both HNF and ciliate abundance were synchronized at T72 h in 10 and 20 µm treatments with 104.3 and 156 ciliates mL^−1^, respectively, just after the sharp bacterial abundance decline (Fig. 3E and F). The bacterivorous scuticociliates *C. glaucoma* (10 µm: 40 cells mL^−1^, 20 µm: 48.4 cells mL^−1^) and *C. margaritaceum* (only in 20 µm: 32.7 cells mL^−1^) and the detritivorous *C. mucicola* (10 µm: 27.4 cells mL^−1^, 20 µm: 23.4 cells mL^−1^) were the prominent species (see Fig. 3E and F). Notably, control treatments (10 µm: 54.8 cells mL^−1^, 20 µm: 68 cells mL^−1^ at T72h) also showed moderate growth responses (Fig. 3B and C). Results of the analysis of variance (ANOVA) revealed that time (F = 33.125, *P* < 0.001), prey amendment (F = 18.288, *P* < 0.001), and size fractionation (F = 6.827, *P* = 0.006) yielded significant effects on ciliate abundance.

### Shift in the composition of ciliate communities: insights from sequence analysis

**Fig 4:**
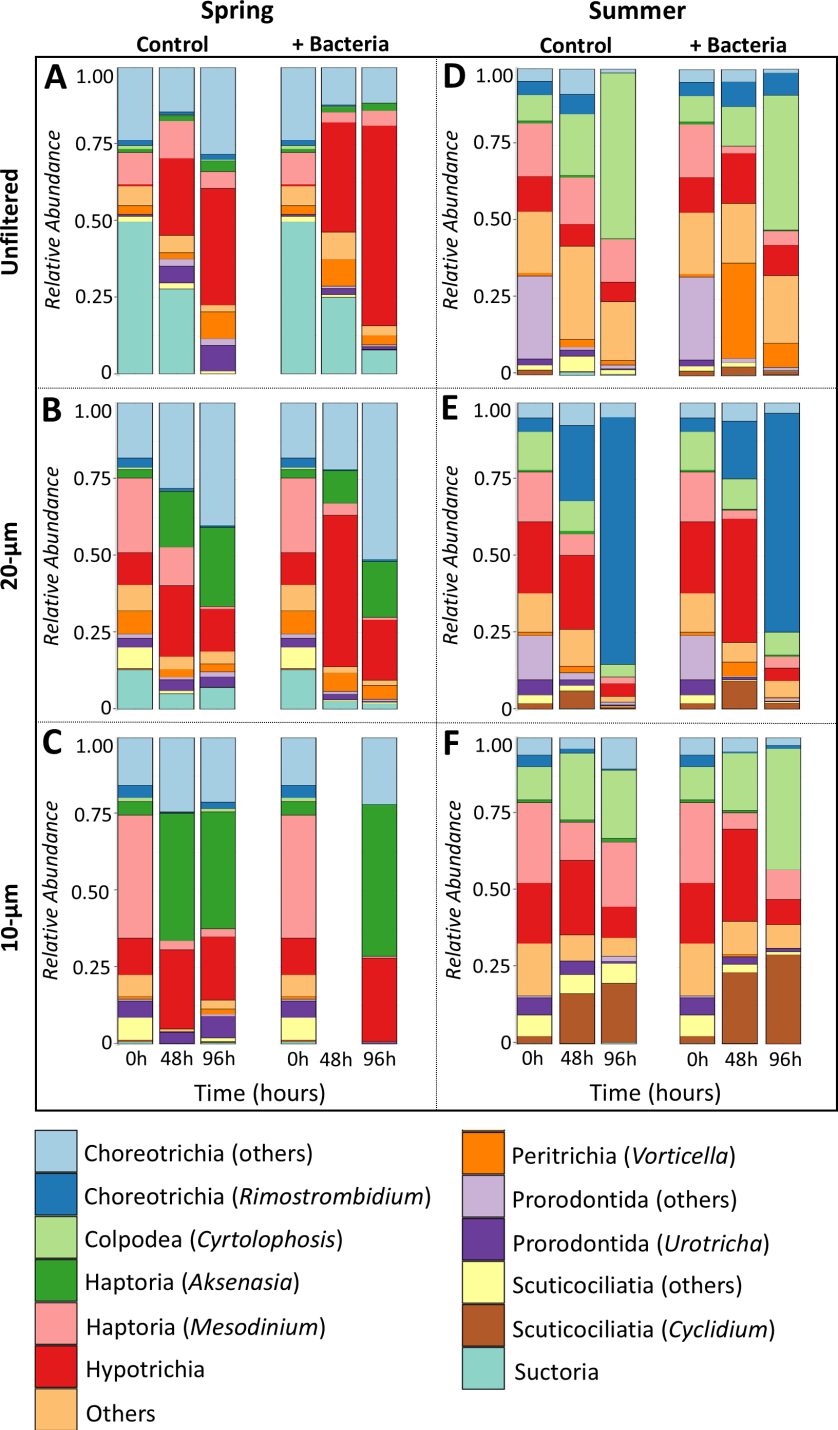
Relative abundance of ciliates in the spring and summer experiments over time in the different size fractions by long-read amplicon sequencing. The results are shown for the respective size fractions in separated columns representing control treatments (Control) and prey-amended treatments (+ Bacteria). To align the sequencing results with microscopic counts, ASV abundance data of microscopically abundant species are presented for respective groups.

After applying the long-read workflow previously established for DADA2, an average of 98.44% ± 0.01% of reads passed all quality filtering steps. A total of 224,506 high-quality reads from the entire eukaryotic community were obtained, distributed across 3,748 amplicon sequence variants (ASVs) with an average of 7,729.01 ± 4,186.16 reads per sample (minimum = 2,727 reads, maximum = 22,248 reads). From the whole eukaryotic community, a subset of 465 ASVs was affiliated with the phylum Ciliophora, comprising 56,846 high-quality reads. Spring samples contained 326 ASVs classified in 10 classes, 15 orders, 26 families, and 32 genera. No sequences from the bacteria-amended 10 µm treatment at 48 h passed the filtering steps, and this sample was not considered for analysis. The dominant groups in the spring ASVs were Hypotrichia (26.1%), Choreotrichia (23.5%), *Askenasia* (16.1%), and *Mesodinium* (9.0%) from the subclass Haptoria (Fig. 4A through C). The unfiltered samples showed high proportions of Suctoria at the starting point, which were later replaced by Hypotrichia (Stichotrichia), especially in the bacteria-amended samples (Fig. 4A). On the other hand, 10 µm samples showed high relative abundance of *Mesodinium* at T0 h (40.1%), replaced by *Askenasia* along the experimental time in both treatments (Fig. 4C). However, both species were microscopically rare. While prostomatid *Urotricha spp*. and *B. planctonicum* were the dominant morphotypes, accounting together for more than 85% of total ciliates observed by QPS (Fig. 3F) but contributed disproportionally less to the sequencing data. Only *Urotricha* accounted for 3.3% of all ASVs, while *B. planctonicum,* together with *P. rostrata*, *C. cratera*, and *C. margaritaceum,* remained undetected by sequence analysis among the 18 genera identified microscopically (Table 2). *Mesodinium* also showed higher diversity through sequence analysis (*M. pulex*, *M. rubrum*, *Mesodinium sp*.). In 20 µm samples, Choreotrichia was the dominant group at T96 h (40.6% in control and 51.1% in bacteria-amended samples, Fig. 4B).

During summer, we detected 345 ASVs assigned to nine classes, 17 orders, 33 families, and 34 genera. Choreotrichia (27.5%), Colpodea (19.8%), and Hypotrichia (13.4%) showed the higher relative abundance (Fig. 4). Microscopically, scuticociliates *Cyclidium, C. margaritaceum,* and colpodean *Cyrtolophosis* showed relatively high abundances in summer (Fig. 3E and F); however, *C. margaritaceum* was undetected in the sequence analysis.

In contrast to spring, prostome ciliates from order Prorodontida (25.5%) were the dominant group at the starting point in the unfiltered samples (Fig. 4D), while haptorian *Mesodinium* dominated the 10 and 20 µm samples (Fig. 4E and F). Later, it was replaced by colpodean *Cyrtolophosis* (>45%) in all bacteria-amended treatments (unfiltered, 10 µm and 20 µm) at T96 h. In bacteria-amended, unfiltered treatment, peritrichous *Vorticella* (32.1%), and in 10 µm, sucticociliate *Cyclidium* showed abundance peak at T96 h (Fig. 4D and F). While five distinct ASVs of *Vorticella* (*V. aequilate*, *V. convallaria*, *V. microstoma*, *V. aquadulcis*, and *V. astyliformis*) were detected, only two species (*V. natans* and *V. aquadulcis* complex) were detected microscopically (Table 2). Notably, only the 20 µm treatment from the control group showed a different trend with a remarkable increase in the proportion of choreotrichous *Rimostrombidium* (81%), which became the dominant group at the experimental end (Fig. 4E). However, microscopically, this genus contributed only to 7% of total ciliate abundance (Fig. 3E).

**Fig 5:**
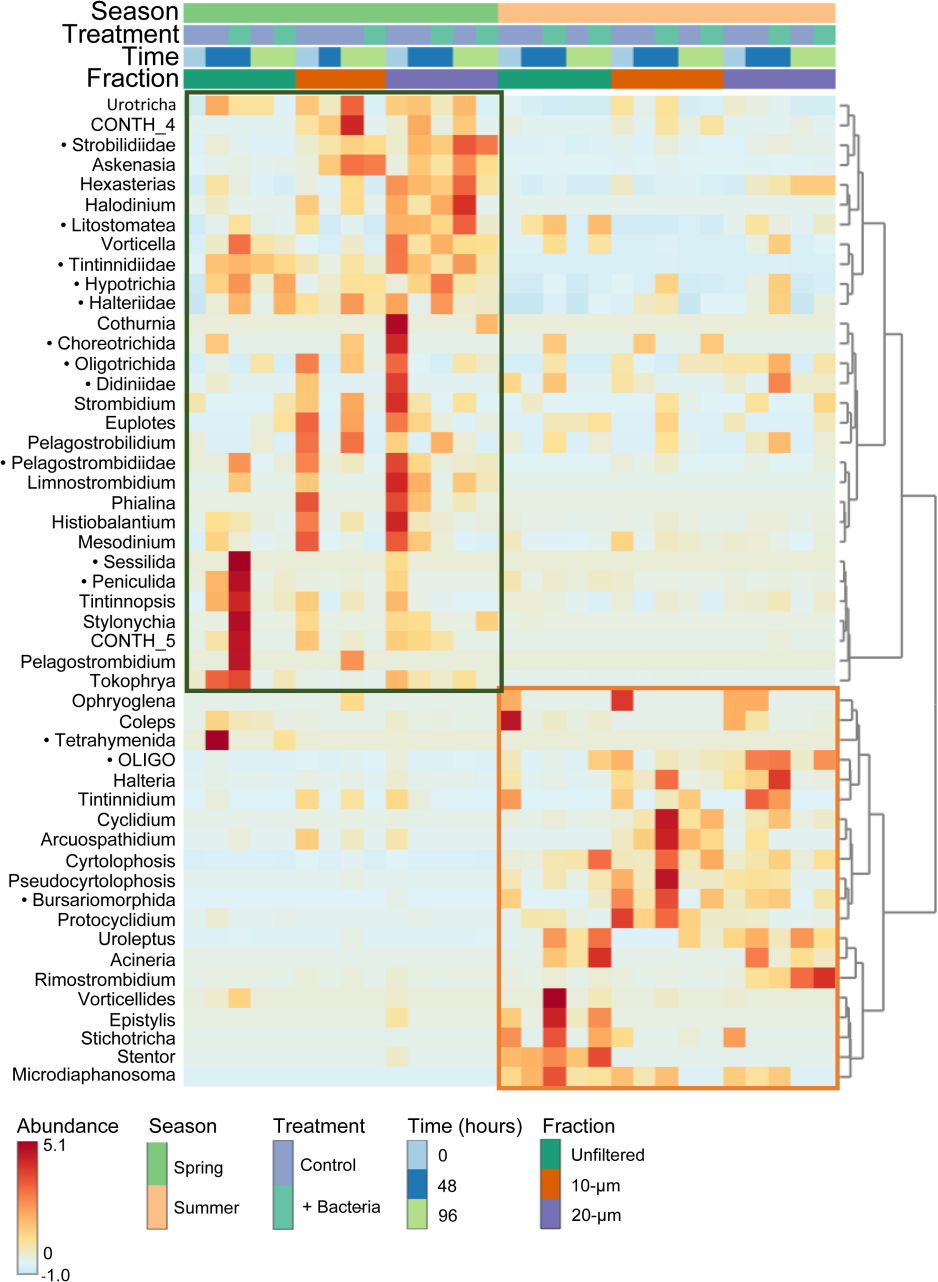
A clustered heatmap showing the variation of relative abundance of the main taxa which distinguishes the samples from different seasons (spring and summer) by long-read amplicon sequencing. Black dots next to the taxa names indicate the unidentified ASVs at genus level.

dbRDA coupled with PERMANOVA identified seasonality as the main explainer of the variance of the samples (35.51% explained, *P* = 0.001) by sequencing, as can also be seen from the PCoA multivariate analysis (File S1; Fig. S2). To identify the main taxonomic markers that distinguish the seasons, we performed hierarchical clustering based on relative abundance. The resulting heatmap clearly highlighted distinct seasonal groupings, with specific taxa showing high relative abundance within each season, thereby accounting for most of the dissimilarity between samples. More taxa stood out as characteristic in spring than in summer, particularly those not identified at the genus level (Fig. 5).

## DISCUSSION

Two initial seasonally different ciliate communities, reflecting season-specific characteristics of food availability, were experimentally manipulated in the same way (Fig. 1) but showed distinct season-specific responses to the experimental manipulations in terms of ciliate abundance and community dynamics.

### Comparison of *in situ* and food web manipulation study during spring

The beginning of spring bloom is usually initiated by the proliferation of small prostome *B. planctonicum*, which peaks immediately at the initiation of phytoplankton bloom with a considerable contribution of phytoflagellates, but then declines, being replaced by larger ciliates with different life strategies (16, 26). In our study, we collected samples during the mid-phase of the spring bloom, which already showed low numbers of *B. planctonicum* (1 cell mL^−1^). In many lakes (e.g., Lakes Constance and Zurich), *B. planctonicum* is replaced by algivorous/predatory *Urotricha* spp., *H. bodamicum,* and *Mesodinium* sp. during the bloom phase (18, 27). However, in our study, bacterial-amended treatments triggered a steep increase of *Urotricha* spp. and *B. planctonicum*. This increase likely reflects the rapidly growing HNF abundance (Fig. 2D through F) as a result of bacterial enrichment, dominated by bacterivorous cryptophytes (28, 29) that likely served as prey for the prostomes. *B. planctonicum* is a typical r-strategist and responds rapidly to enhanced cryptophyte prey (18, 26, 27) and together with *Urotricha* spp. is a typical flagellate predator (26, 28). Indeed, the abundance of HNF peaks in spring plankton, followed by the peak of prostomes (16, 18), further confirming our assumption about the complexity of trophic cascade from HNFs to small raptorial ciliates (30).

Several studies have attributed the mid-spring decline in ciliate communities, prior to the onset of the clear-water phase, to predation by large grazers such as copepods (calanoids and cyclopoids), cladocerans, and rotifers (19, 31, 32). Many studies (e.g., 33–35) observed that ciliate communities are subject to strong top-down control by mesozooplankton, especially copepods, followed by intraguild predation by daphnids and food competition, which eventually leads to the dramatic decline of ciliate communities. Similarly, mesozooplankton, including cladocerans, copepods, and rotifers, showed their annual maximum during spring bloom at our sampling site (17, 36).

Notably, we also observed a strong top-down control in our spring experiment, particularly in unfiltered treatments where ciliates were decimated by large zooplankton, resulting in a dramatic drop to negligible ciliate numbers towards the experimental end (Fig. 2A and D), thus confirming general trends described for late clear water phase (11, 37). Surprisingly, the grazing control of zooplankton even overrode the busting effect of the considerable bacterial amendment and thus did not induce any net ciliate growth as compared with the marked growth response of HNF (Fig. 2D). However, in the filtered treatments (20 µm and 10 µm), ciliate communities were released from the grazing pressure and increased rapidly, especially in bacterial amended treatments, with approximately daily doubling, comparable to the growth rates typical for microbes in pelagic environments (18, 28). Also, the size fractionation had a highly significant effect on ciliate abundance and species composition in spring. Thus, we conclude that the strong top-down control during the bloom phase considerably shaped the ciliate community in our treatments (5, 11, 13, 26, 34, 37). The trends nicely link the outcomes of experiments with the seasonal studies.

### Comparison of *in situ* and food web manipulation study during summer

In previous studies conducted in Římov during the summer (24, 38), ciliates consumed ~20% of total bacterial production. Ciliate communities were dominated by small-sized (<25 µm) species, especially *H. grandinella*, which was later replaced by *Pelagostrobilidium*, *Coleps* sp., and *C. mucicola*, while *Urotricha* spp. and *C. margaritaceum* made stable contributions (24). We observed similar patterns in ciliate communities in our summer experiment (Fig. 3A through F). Notably, the omnivorous/bacterivorous *H. grandinella* was the dominant species in *in situ* studies and an important omnivorous species linking different trophic levels (24, 39, 40); however, we observed only slight increases in our experiments. The enhanced bacterial prey availability likely supported bacterivorous/detritivorous species, as reflected by the observed dominance of *C. glaucoma, C. margaritaceum,* and *C. mucicola* (Fig. 3D through F).

In all bacteria-amended treatments, *C. glaucoma* was a dominant species (Fig. 3D through F), underlying that *Cyclidium* is an efficient bacterivore (24), which is known to be size-selective when feeding on morphologically diverse bacterial communities (41–43). Moreover, our results clearly reflected the positive cascading effect of bacterial enrichment on the total ciliate abundance. Besides, the common maxima of both HNF and ciliate abundance were recorded at T72 h, just after the sharp decline of bacterial numbers (Fig. 3D through F), indicating the direct transfer of energy from bacteria to both these bacterivorous protistan groups.

Generally, grazing pressure of larger zooplankton is assumed to be weaker during the summer season (11, 17), and thus ciliate communities should be subjected to a weaker top-down control of large zooplankton. Our experimental manipulations also suggest weaker effects of summer grazer community on ciliate abundance. For instance, the results from unfiltered treatments indicated moderate levels of grazing pressure on protists, as evidenced by the significantly higher ciliate abundance in unfiltered treatments during summer (Fig. 3A and D) as compared with the unfiltered treatment during spring (Fig. 2A and D).

Ecological studies on ciliates have been carried out both *in situ* and in the laboratory (13, 28, 39, 44). *In situ* experiments mimic natural conditions well but encounter difficulties when conducting experiments on complex planktonic systems, resulting in a lack of precision (45, 46). On the other hand, manipulation of natural plankton communities in the laboratory eliminates the effects of various interfering factors. However, it offers the possibility to accurately measure impacts of specific factors (47, 48), in our case the effects of food availability and top-down control by grazers. Despite some possible shortcomings of the experimental manipulations (49), our study demonstrates its usefulness for providing valuable information on the trophic cascading and top-down control in the seasonally differently shaped microbial communities (48, 50).

### High-throughput sequencing-based results reflect higher ciliate diversity

Sequencing-based results generally provide a more comprehensive picture of ciliate diversity (21, 51–53). In a study on the composition of ciliates in the Krka River, 214 ciliate genera were detected by molecular studies, while only 26 genera were identified microscopically (54). Similarly, only 30 ciliate genera were observed microscopically, and 47 were detected by molecular studies in a mountain lake (55). In line with the previous studies, our sequencing data also revealed a much broader ciliate diversity with specific sequences detected in both experiments (Fig. 5). However, only 32 ASVs in spring and 36 in summer could be assigned to genus level, which indicates a huge hidden diversity in ciliates.

Numerous studies have also reported large discrepancies between microscopic and molecular abundance data (21, 56, 57). The possession of extremely high copy number, taxonomically uneven distribution, and sequence polymorphism of the 18S rRNA genes are among the major reasons for the observed discrepancy (58, 59). Due to a very high copy number of their 18S rRNA gene, ciliate-related reads usually dominate the molecular analysis of environmental samples, but their high-read abundance is usually poorly supported by morphological studies (54, 60, 61). This highlights the obstacle of using only the sequence-based approaches to determine the abundance of ciliates (57, 59).

Our study confirmed the incongruencies in relative abundance of ciliates between morphological and sequence-based approaches. During spring, Hypotrichia, Choreotrichia, Haptoria (*Aksenasia* and *Mesodinium*), and during summer, Choreotrichia, Colpodea, and Hypotrichia dominated the sequencing results (Fig. 4) but contributed far less to microscopic counts (55, 57, 60), except for Colpodea (*Cyrtolophosis*), which was a dominating morphotype during summer (Fig. 3D and F). An obvious reason for this is the extremely high copy number of rRNA genes in ciliates, with the real extremes being reported for the hypotrichous *H. grandinella* (567,893 copies) and in peritrichous (e.g., *Vorticella* with 316,000 copies) ciliates (59, 62). Not surprising then, *Vorticella* accounted for <1% of the total number of ciliates in bacteria-amended unfiltered treatment at T48 h in summer but accounted for more than 32% of all sequences (Fig. 4D) and Hypotrichia (*Halteria*) accounted for 26.1% of total ASVs during spring and 13.4% during summer. Similarly, Choreotrichia (*Rimostrombidium*) also have high gene copy numbers (58–60). While they represented <10% of cell counts, they accounted for >80% of all sequences in 20 µm control treatment at T96 h during summer (Fig. 4E). However, Prostome ciliates have relatively low copy numbers (e.g., *Coleps*: 2,100–5,800) (58), resulting in their low representation in the sequencing data (Fig. 4A through C), despite being microscopically the most abundant group during spring (Fig. 2). Interestingly, we found that our molecular data of Scuticociliatia (*Cyclidium*) and Colpodea (*C. mucicola*) projected similarities with the microscopic counts, which corroborated previous studies (21, 63). This is most likely due to lower copy numbers observed in Scuticociliatia (~22,100) (58) and Colpodea (~19,800) (64). Our results suggest that sequencing-based analyses could only provide a rough estimate of the abundance of ciliates with lower copy numbers of rRNA genes but are unreliable for ciliates with high copy numbers. Therefore, for ecological studies in ciliates, sequence-based results should be accompanied by classical morphological analysis such as QPS.

However, qualitative sequence-based analysis (presence/absence) is particularly useful for complex environments where it is difficult to collect samples for traditional morphological methods. The results are especially useful for comparing different environments and seasons in terms of community composition (Fig. 5) and the distribution of different trophic and functional groups (65, 66). They also play an important role in the study of phylogenetic signals to understand how ecological traits are related to the evolutionary relationship between different ciliate species (67). Such methods show greater diversity and are particularly useful for the identification of morphologically indistinguishable species, which usually form a cryptic species complex and cannot be identified by only morphological methods, e.g. genera such as *Paramecium* and *Balanion* (68, 69).

Although sequence-based analysis provides a comprehensive overview of community diversity (56, 57, 70, 71), several challenges remain. These include mainly incomplete and error-prone reference databases (54, 55) and lack of ciliate sequences from cultured representatives (21, 55). Consequently, our results were also influenced by these factors. Seven species were undetected in our molecular analysis, specifically, *B. planctonicum* in spring and *C. margaritaceum* in summer, although they clearly dominated the microscopic counts. This discrepancy is probably due to primer bias or the presence of cryptic species complexes for which there are no representative sequences in the public databases (21). On the other hand, bleed-through contamination is also a challenge for sequencing-based analyses (72, 73), especially in studies involving filtration (74). This is because some more fragile ciliate cells can break during the filtration process or pass through the filter pores due to the suction pressure (44). Despite the use of gravity gentle filtration, we also observed the sequences of several larger ciliates and zooflagellates in our data, even in 10-μm and 20-μm treatments. Usually, it is not the cell length, but the cell width, that decides if the species can pass through a filter of a defined porosity.

Thus, we conclude that despite these limitations, sequencing-based analysis is very effective in revealing the cryptic diversity of ciliates and in processing a large number and volume of samples even from those environments that are difficult to study using purely morphological techniques. Moreover, the long-read sequencing technique used in this study allowed us to obtain longer sequences, which provided more accurate taxonomic classifications. In our study, we found clear seasonality among ciliate communities with respect to the sequence abundance of distinct groups (Fig. 5). This highlights the importance of the usage of molecular techniques in ecological studies, and ideally when complemented with microscopic techniques. Therefore, the combination of novel sequencing approaches with classical microscopy techniques is still quite essential. This is because it enables deeper and more precise insights into the diversity and ecological aspects of ciliates, such as feeding modes related to preferred prey items and its seasonality, community dynamics, and rapid changes in species abundance under different experimental regimes and seasonal settings.

## MATERIALS AND METHODS

### Study area and sampling site

Samples were collected from a deep, meso-eutrophic Římov Reservoir (48°51N, 14°29E), located south of České Budějovice, Czech Republic. The reservoir is dimictic with a total surface area of 210 ha and the maximum and mean depth of 42 m and 16 m, respectively (75, 76). Samples for two experiments were taken in the spring (19 April) and summer (22 August) of the year 2022 from the epilimnion of the dam area, representing the deepest part of the reservoir. Vertical profiles of temperature, pH, and dissolved oxygen (DO) concentration were measured at the sampling sites by a YSI EXO II multiparameter probe (Yellow Springs Instruments, USA).

### Sampling and experimental manipulation

Forty liters of water was taken from the epilimnion (0.5 m depth) in spring and from a slightly deeper layer (4 m depth) in summer, due to the low DO concentration in the surface layer covered by cyanobacteria. The samples were collected by Friedinger sampler (Šramhauser spol. s.r.o., Czech Republic). Two liters of an untreated subsample was separated for further analysis of chemical parameters including chlorophyll-*a* (Chl-*a*), total organic carbon (TOC), total nitrogen, and dissolved phosphorus (DP) (77).

To test for trophic cascading effects of different grazers, we manipulated top-down controlling factors using the size-fractionation approach similar to that described elsewhere (28). Size fractionation was done by gravity filtration through 20 µm and 10 µm pore-size filters (diameter 142 mm, Sterlitech, USA) and yielded treatments of different food web complexity (Fig. 1): (i) the 10 µm fraction contained mainly prokaryotes, small flagellates (<10 µm), and nano-sized ciliates (<15 µm); (ii) a more complex 20 µm fraction, which contained additionally also micro-sized ciliates (≥15–30 µm) and large-sized flagellates (10–20 µm), and (iii) compared with an unfiltered whole water sample serving as control with complex natural plankton community. This experimental setup allowed releasing the microbial communities from grazing pressure by larger zooplankton or larger protists, thus gradually representing more and more simplified microbial food webs; from unfiltered to the 20 µm and 10 µm filtered treatments. The water from all the size fractions was collected in 2 L bottles, and a total of 6 bottles (12 L) were prepared for each treatment (Fig. 1).

In addition, to manipulate bottom-up factors aimed at accelerating trophic cascading within the microbial food webs, one half of all the types of triplicate treatments were amended by pre-cultured bacterial prey (a mixture of isolated strains *Limnohabitans planktonicus* and *L. parvus*) isolated from the reservoir. These bacteria have been proven to be suitable food resources boosting the growth of natural communities of bacterivorous protists (28, 78). The amount of the added bacteria was ca. 5–6 times more than the natural background bacterial abundance (yielding the final concentration of 15–20 × 10^6^ cells mL^−1^). All treatments were incubated in the dark at 16°C to set a comparable growth potential of ciliates in both experiments conducted over a period of 96 h, and subsamples of defined volume were aseptically taken every 24 h at a clean bench.

### Enumeration of HNFs and bacteria

Fifteen to twenty milliliter sub-samples were daily collected from each triplicate and fixed with formaldehyde (2% final concentration). The total bacterial cell counts were quantified via flow cytometry in samples stained with SYBR Green using a CytoFLEX S flow cytometer (Beckman Coulter), equipped with a blue laser and bandpass filters 525/40 and 690/50. For the enumeration of HNFs, 5–10 mL subsamples were stained with DAPI (4′,6-diamidino-2-phenylindole, final concentration of 1 µg mL^−1^) and filtered onto 1 µm pore-sized black polycarbonate membrane filters (Sterlitech, USA), and total HNFs were counted under an epifluorescence microscope at a magnification of 1,000× according to reference 28.

### Enumeration and identification of ciliates

Samples (100 mL) were daily collected from all triplicate treatments and divided into two 50 mL subsamples. One 50 mL subsample was fixed with formaldehyde (2% final concentration), stained with DAPI, and filtered onto black 1 µm pore-sized polycarbonate membrane filters (Sterlitech, USA) for total ciliate counts and identification via epifluorescence microscopy (5, 28, 39, 42). For taxonomic resolution of the morphotypes, we used quantitative protargol staining method (QPS) with second 50 mL subsample that was fixed with Bouin’s solution (5% final concentration) and filtered onto 0.8 µm pore-size nitrocellulose filters (Sartorius, Germany) and stained following the protocol of O. Skibbe (79), with modifications suggested elsewhere (80). Filters were inspected under bright-field microscopy (Olympus BX50; Japan). Cells were taxonomically identified by using the taxonomic keys of Foissner et al. (81–83) and W. Foissner and H. Berger (84)

### DNA extraction, PCR amplification, and PacBio sequencing

Three subsamples were collected through the course of the experiment at times 0, 48, and 96 h. A 350 mL subsample was taken from each replicate, and the triplicates from the individual treatments were pooled, resulting in a total volume of 1,050 mL per treatment. Biomass was collected on 0.22 µm polyether sulfone membrane filters (MILLIPORE EXPRESS, Germany) and stored with 1 mL of DNA/RNA Shield at −80°C. For long-read sequencing, DNA was extracted with the Quick-DNA High Molecular Weight MagBead Kit (Zymo Research, USA) according to the manufacturer’s instructions, and the concentration was quantified using a Qubit fluorometer (Invitrogen, USA). Extracted DNA was sent to Rush University Genomics and Microbiome Core Facility (Chicago, USA), and there, the amplification was conducted with the primer pairs EukV4F (56) and Euk21R (85) to amplify the 18S rRNA, ITS1, ITS2 and a part of the 28S gene following the previously established protocol by Latz et al. 2022 (86). An equimolar library was prepared using Fluidigm barcodes, followed by PacBio library preparation with the SMRTbell system for high-fidelity long-read sequencing. The sequencing was performed on a Sequel II platform using a SMRT 8 M cell with a 30-h movie runtime. The resulting sequences were demultiplexed and dereplicated at the Rush University Research Bioinformatics Core Facility using DADA2 script specifically designed for PacBio long-amplicon data (87). Chimeric sequences were identified and filtered using the BimeraDenovo algorithm, with a minimum fold parent-over-abundance threshold set to 3.5. Generated long-read sequences were assessed to further quality filtering, primer removal, trimming, and denoising using the DADA2 R package, largely following the long-read workflow previously established for the same approach (86, 87). The 18S rRNA gene database PR2 version 5.0.1 (88) was used as a training set for taxonomic classification with assignTaxonomy of DADA2. Sequencing data generated in this study are available at the European Nucleotide Archive (ENA) under the BioProject number PRJEB86180.

### Statistical analysis

An ANOVA was performed to examine the effects of time, size fraction, and prey amendment (explanatory variables) on ciliate abundance. For long-read amplicon sequences, all amplicon sequence variants (ASVs) belonging to the group Ciliophora were considered for further analysis in RStudio (89) and MicrobiomeAnalyst (90). Data were pre-processed to ensure data equality, where taxa were retained if they were present (non-zero counts) in at least 10% of the samples, as a prevalence threshold, and a centered log-ratio (CLR) transformation was applied to normalize the compositional nature of the data, as recommended by reference 91. Diversity indices were evaluated using the “vegan” package (92) and a two-way analysis of variance (ANOVA) for variation in the Shannon diversity index with factors being treatments (control or bacterial amended), size fraction (unfiltered, 10 µm, 20 µm), and different time points (0, 48, 96 h). We performed the post-hoc Tukey’s honestly significant difference (HSD) test on significant results of the ANOVA to identify which specific groups differed significantly from each other. The β-diversity comparison was performed by principal coordinate analysis (PCoA) using Bray-Curtis dissimilarity and a hierarchical clustering ordination based on the relative abundance of the main genus on the grouping factor was visualized by heatmap using Ward algorithm. Distance-based redundancy analysis (dbRDA) was used to evaluate the dissimilarity between the samples using miaverse package (93) and the permutational ANOVA (PERMANOVA) estimated the statistical significance of the differences in community composition and the variation explained by each grouping factor (treatments, size fraction, and time points) using adonis2 function from the vegan package. The R codes used for statistical analysis and creating figures are included in File S2.

## Data Availability

Sequencing data generated in this study are available at the European Nucleotide Archive (ENA) under the BioProject number PRJEB86180. Supporting figures and scripts are available at https://doi.org/10.6084/m9.figshare.29521199.v1.

## SUPPLEMENTAL MATERIAL

File S1 msystems.00304-25-s0001.docx Figures S1 and S2.

File S2 msystems.00304-25-s0002.docx R codes used for statistical analysis and creation of figures.

ASM does not own the copyrights to Supplemental Material that may be linked to, or accessed through, an article. The authors have granted ASM a non-exclusive, world-wide license to publish the Supplemental Material files. Please contact the corresponding author directly for reuse.
