# Supplementary material for: Morphological and molecular analyses of season-specific responses of freshwater ciliate communities to top-down and bottom-up experimental manipulations
Source: mSystems. 2025 Aug 15;10(9):e00304-25. doi: 10.1128/msystems.00304-25 (PMC12455964; doi:10.1128/msystems.00304-25)
Supplement: File S1 — Figures S1 and S2. [file msystems.00304-25-s0001.docx]

**SUPPLEMENTARY MATERIAL**

Figures

# Morphological and molecular analyses of season-specific responses of freshwater ciliate communities to top-down and bottom-up experimental manipulations

Usman Asghar^1,2^, Indranil Mukherjee^1^#, Bettina Sonntag^3^, Caio César Pires de Paula^1^, Vojtěch Kasalický^1^, Paul-Adrian Bulzu^1^, Anusha Priya Singh^1,2^, Tanja Shabarova^1^, Kasia Piwosz^4^, Karel Šimek^1^

*^1^Biology Centre of the Czech Academy of Sciences, Institute of Hydrobiology, Na Sádkách 7, 37005, České Budějovice, Czech Republic*

*^2^Faculty of Science, University of South Bohemia, 37005, České Budějovice, Czech Republic*

*^3^Research Department for Limnology, Mondsee, Universität Innsbruck, A-5310 Mondsee, Austria*

*^4^National Marine Fisheries Research Institute, ul. Kołłątaja 1, 81-332 Gdynia, Poland*

**# Correspondence: Indranil Mukherjee**

Biology Centre CAS, Institute of Hydrobiology

Na Sádkách 7, 37005 České Budějovice, Czech Republic

Telephone number: +420 387775873

FAX number: +420 385310248

E-mail: indranilmukherjee04@yahoo.com


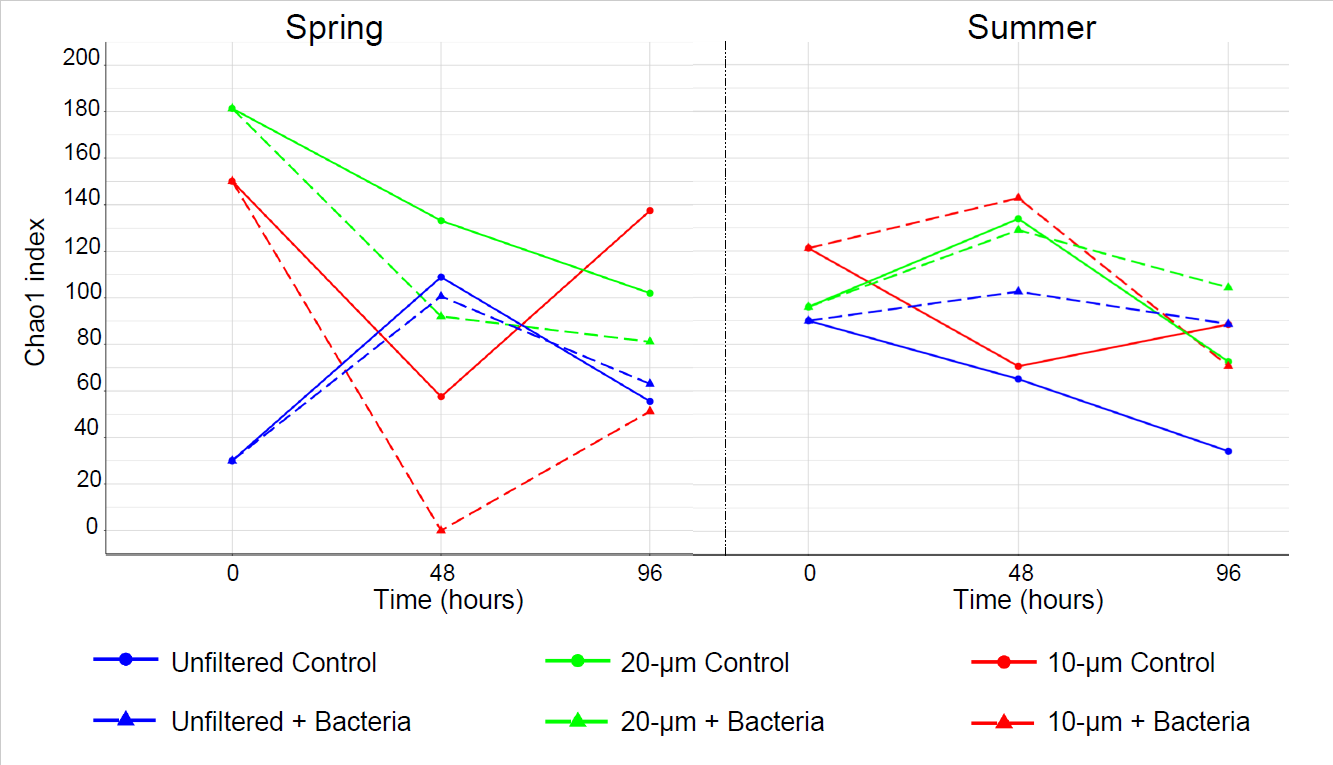


**Figure S1.** Diversity index (Shannon-Wiener) comparing long-read amplicon sequencing result for spring and summer samples over time among the different fraction sizes (blue = unfiltered, red = 10 μm, green = 20 μm). The samples were separated into a control group (solid line) and prey-amended treatment (dashed line).

**
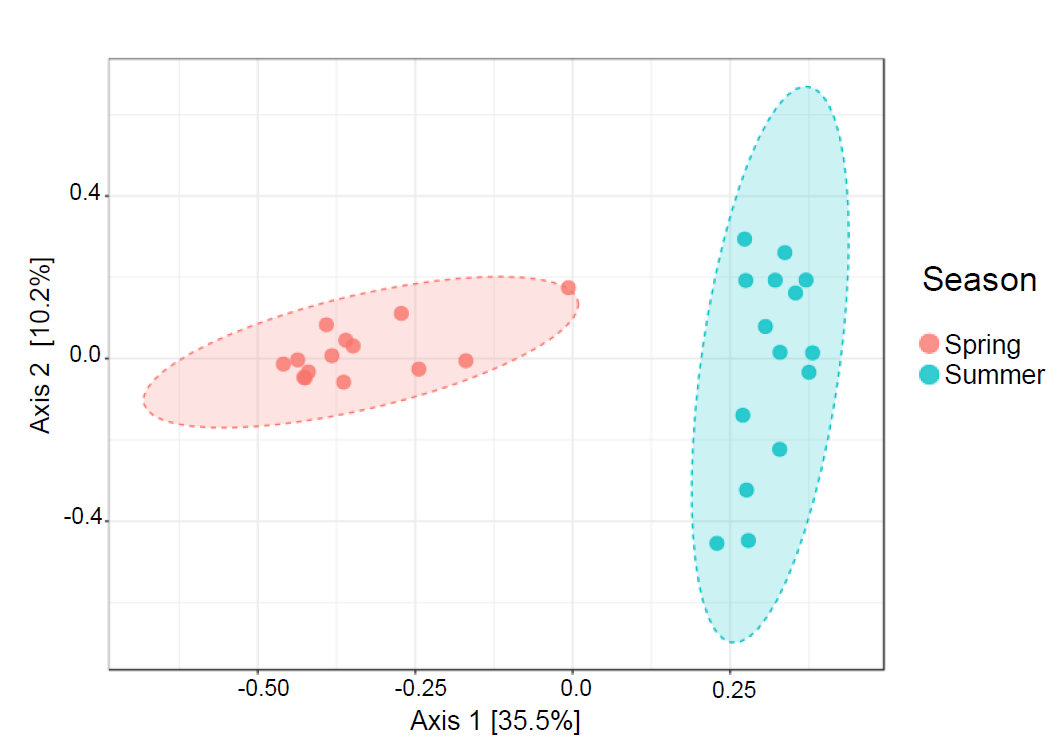
**

**Figure S2.** Principal Coordinates Analysis (PCoA; Bray-Curtis distance) plot visualizing the ß-diversity of the ciliate communities in the context of seasonal changes (spring and summer) by long-read amplicon sequencing. Ellipses indicate 95% confidence interval. Each dot represents the time-point sampling along the experimental design.
