# Supplementary material for: Morphological and molecular analyses of season-specific responses of freshwater ciliate communities to top-down and bottom-up experimental manipulations
Source: mSystems. 2025 Aug 15;10(9):e00304-25. doi: 10.1128/msystems.00304-25 (PMC12455964; doi:10.1128/msystems.00304-25)
Supplement: File S2 — R codes used for statistical analysis and creation of figures. [file msystems.00304-25-s0002.docx]

**SUPPLEMENTARY MATERIAL**

R scripts

# Morphological and molecular analyses of season-specific responses of freshwater ciliate communities to top-down and bottom-up experimental manipulations

Usman Asghar^1,2^, Indranil Mukherjee^1^#, Bettina Sonntag^3^, Caio César Pires de Paula^1^, Vojtěch Kasalický^1^, Paul-Adrian Bulzu^1^, Anusha Priya Singh^1,2^, Tanja Shabarova^1^, Kasia Piwosz^4^, Karel Šimek^1^

*^1^Biology Centre of the Czech Academy of Sciences, Institute of Hydrobiology, Na Sádkách 7, 37005, České Budějovice, Czech Republic*

*^2^Faculty of Science, University of South Bohemia, 37005, České Budějovice, Czech Republic*

*^3^Research Department for Limnology, Mondsee, Universität Innsbruck, A-5310 Mondsee, Austria*

*^4^National Marine Fisheries Research Institute, ul. Kołłątaja 1, 81-332 Gdynia, Poland*

**# Correspondence: Indranil Mukherjee**

Biology Centre CAS, Institute of Hydrobiology

Na Sádkách 7, 37005 České Budějovice, Czech Republic

Telephone number: +420 387775873

FAX number: +420 385310248

E-mail: indranilmukherjee04@yahoo.com

## -----------------------------------------------

## SCRIPT FOR COMPOSITIONAL BARPLOT, DIVERSITY, dbRDA & PERMANOVA

## Description: This script generates compositional barplots from a pivot table,

## calculates Shannon and Chao1 diversity indices, tests differences with ANOVA,

## and performs dbRDA and PERMANOVA to test effects of metadata factors.

## -----------------------------------------------

## Load required packages ##

library(tidyverse)

library(ggplot2)

library(dplyr)

library(tidyr)

library(RColorBrewer)

library(phyloseq)

library(microbiome)

library(vegan)

library(mia)

library(miaViz)

library(MultiAssayExperiment)

library(ggpubr)

library(knitr)

## -----------------------------------------------

## SECTION 1: COMPOSITIONAL BAR GRAPH

## -----------------------------------------------

# Load OTU composition table (Orders in rows, samples in columns)

data <- read.table("Order data.txt", header = TRUE, sep = "\t", dec = ".")

# Convert to long format for ggplot

data_long <- data %>%

pivot_longer(cols = -Order, names_to = "SamplePoint", values_to = "Proportion")

# Define a custom color palette

my_colors <- c(

"#a6cee3", "#1f78b4", "#b2df8a", "#33a02c", "#fb9a99",

"#e31a1c", "#fdbf6f", "#ff7f00", "#cab2d6", "#6a3d9a",

"#ffff99", "#b15928", "#8dd3c7"

)

# Generate the proportional bar plot

ggplot(data_long, aes(x = SamplePoint, y = Proportion, fill = Order)) +

geom_col(position = "fill", color = "black", size = 0.2) +

scale_y_continuous(labels = scales::percent_format(scale = 1)) +

scale_fill_manual(values = my_colors) +

labs(title = "Proportion of Orders Across Sample Points",

x = "Sample Point",

y = "Proportion (%)",

fill = "Order") +

theme_minimal() +

theme(

axis.text.x = element_text(angle = 45, hjust = 1, size = 10),

axis.title = element_text(size = 12, face = "bold"),

plot.title = element_text(size = 14, face = "bold")

)

# Save plot to TIFF

ggsave("Proportional_Orders.tiff", width = 12, height = 8, dpi = 600,

units = "in", device = "tiff", compression = "lzw")

## -----------------------------------------------

## SECTION 2: DIVERSITY INDICES AND ANOVA

## -----------------------------------------------

# Load OTU, taxonomy, and metadata tables

OTU <- read.table("ASV table ciliophora.txt", header = TRUE, row.names = 1)

taxmat <- read.table("tax table ciliophora.txt", header = TRUE, row.names = 1)

metadata <- read.table("map file ciliophora.txt", header = TRUE, row.names = 1)

# Create phyloseq object

physeq <- phyloseq(

otu_table(as.matrix(OTU), taxa_are_rows = TRUE),

tax_table(as.matrix(taxmat)),

sample_data(metadata)

)

# Transform to compositional

pseq.rel <- microbiome::transform(physeq, "compositional")

# Subset to core taxa (>=0.1% abundance, in >=10% samples)

pseq.core <- core(physeq, detection = 0.001, prevalence = 0.1)

# Calculate alpha diversity (Chao1, Shannon, etc.)

alpha_div <- alpha(pseq.core, index = "all")

sample_metadata <- as.data.frame(sample_data(physeq))

sample_metadata$Chao1 <- alpha_div$chao1

sample_metadata$Shannon <- alpha_div$shannon

# Test Chao1 normality - Run the same for Shannon diversity index

shapiro.test(sample_metadata$Chao1)

# Boxplot with group comparison

ggviolin(sample_metadata, x = "Season", y = "Chao1", fill = "Season",

palette = "Set3", add = "boxplot") +

stat_compare_means(method = "anova")

## -----------------------------------------------

## SECTION 3: dbRDA AND PERMANOVA

## -----------------------------------------------

# Convert phyloseq to TreeSummarizedExperiment (TSE)

tse <- makeTreeSummarizedExperimentFromPhyloseq(physeq)

# Apply transformations

tse <- transformAssay(tse, assay.type = "counts", method = "relabundance")

tse <- transformAssay(tse, assay.type = "relabundance", method = "clr", pseudocount = TRUE)

# Add divergence from median profile

tse <- addDivergence(tse, assay.type = "counts", reference = "median", FUN = getDissimilarity)

# Run dbRDA with Bray-Curtis dissimilarity

tse2 <- addRDA(

tse,

assay.type = "relabundance",

formula = assay ~ Season + Bacteria + Day + Fraction,

distance = "bray",

na.action = na.exclude

)

# Extract PERMANOVA table

rda_info <- attr(reducedDim(tse2, "RDA"), "significance")

kable(rda_info$permanova)
